# LRG1 as a novel therapeutic target in eye disease

**DOI:** 10.1038/s41433-021-01807-4

**Published:** 2022-01-05

**Authors:** Giulia De Rossi, Marlene E. Da Vitoria Lobo, John Greenwood, Stephen E. Moss

**Affiliations:** Present Address: Institute of Ophthalmology, University College London, 11-43 Bath Street, London, EC1V 9EL UK

**Keywords:** Antibody therapy, Predictive markers, Neuroscience, Mechanisms of disease

## Abstract

Retinal and choroidal diseases are major causes of blindness and visual impairment in the developed world and on the rise due to an ageing population and diabetes epidemic. Standard of care is centred around blockade of vascular endothelial growth factor (VEGF), but despite having halved the number of patients losing sight, a high rate of patient non-response and loss of efficacy over time are key challenges. Dysregulation of vascular homoeostasis, coupled with fibrosis and inflammation, are major culprits driving sight-threatening eye diseases. Improving our knowledge of these pathological processes should inform the development of new drugs to address the current clinical challenges for patients. Leucine-rich α-2 glycoprotein 1 (LRG1) is an emerging key player in vascular dysfunction, inflammation and fibrosis. Under physiological conditions, LRG1 is constitutively expressed by the liver and granulocytes, but little is known about its normal biological function. In pathological scenarios, such as diabetic retinopathy (DR) and neovascular age-related macular degeneration (nvAMD), its expression is ectopically upregulated and it acquires a much better understood pathogenic role. Context-dependent modulation of the transforming growth-factor β (TGFβ) pathway is one of the main activities of LRG1, but additional roles have recently been emerging. This review aims to highlight the clinical and pre-clinical evidence for the pathogenic contribution of LRG1 to vascular retinopathies, as well as extrapolate from other diseases, functions which may be relevant to eye disease. Finally, we will provide a current update on the development of anti-LRG1 therapies for the treatment of nvAMD.

## 摘要

在发达国家, 视网膜和脉络膜疾病是失明和视力损害的主要原因, 而且由于人口老龄化及糖尿病盛行, 这一疾病的发病率正在上升。标准治疗是以阻断血管内皮生长因子 (vascular endothelial growth factor, VEGF) 为核心, 但尽管失明患者数量减少了一半, 但随着时间的推移, 高比率的患者无反应和疗效丧失是关键挑战。血管稳态失调, 伴有纤维化和炎症, 是导致威胁视力的眼病的主要元凶。提高我们对这些病理过程的认识有助于开发新药, 以解决目前患者面临的临床挑战。富亮氨酸α-2糖蛋白1 (Leucine-rich α-2 glycoprotein 1, LRG1) 作为一个新兴标志物, 在血管功能障碍, 炎症和纤维化方面起着关键作用。在生理条件下, LRG1由肝脏和粒细胞组成表达, 但其正常的生物学功能尚不清楚。在病理情况下, 如糖尿病视网膜病变 (diabetic retinopathy, DR) 和新生血管性年龄相关性黄斑变性 (neovascular age-related macular degeneration, nvAMD), 其表达异位上调, 且致病作用更清楚。LRG1的主要活性之一是对转化生长因子β (Transforming growth-factor β, TGFβ) 通路的环境依赖调节, 但最近又发现新的作用。本综述旨在强调LRG1在血管性视网膜病变中的致病作用的临床和临床前证据, 以及从其他疾病中推断可能与眼病相关的功能。最后, 我们将提供抗LRG1治疗nvAMD的最新进展。

## Introduction

There are currently 2 million people in the UK either legally blind or living with sight-threatening pathologies and this number is predicted to double by 2050. The estimated prevalence of the predominant eye diseases, AMD, glaucoma, cataract and DR is vast with 600,000, 500,000, 500,000 and 144,000 patients respectively [1, 2]. These increases are of course not restricted to the UK but are mirrored globally, rendering this a major international challenge. Alarmingly, the increase in life span together with diabetes and obesity reaching endemic proportions will create a perfect storm for a surge in AMD and DR patients who will require long-term care and treatments [1, 2].

Although disorders of the retina, or retinopathies, are distinct pathologies, many share several common disease hallmarks such as vascular dysfunction (oedema and/or angiogenesis), inflammation, and fibrosis (or extracellular matrix remodelling). Thus, unsurprisingly, such pathologies also share some common molecular drivers. One key molecule is vascular endothelial growth factor (VEGF), which has attracted huge attention in the past 50 years, for its central role in DR, diabetic macular oedema (DMO) and nvAMD [3–6]. VEGF was originally identified as a vascular permeability factor, and was later shown to be a potent mitogen for endothelial cells (ECs) with an ED_50_ of 2–10 picomolar [7]. Through its receptors VEGFR1-3, VEGF can promote EC survival, migration, proliferation and junctional remodelling and, by doing so, it is the master regulator of hyper-permeability responses and angiogenesis. Thus, unsurprisingly, extensive work has been dedicated to disarming VEGF in diseases where vascular leakage and angiogenesis are featured [8]. The treatment of some retinopathies was, in fact, revolutionised by the approval in 2004 of Macugen (Eyetech Inc.), the first anti-VEGF agent and the first aptamer to be licensed for clinical use, and subsequent anti-VEGF drugs including Lucentis (Genentech), Eylea (Regeneron Pharmaceuticals) and Avastin (Genentech, off label), administered at slightly different dosage regimens (once a month, on average) by intra-vitreal (IVT) injection. Collectively these agents have decreased the number of patients becoming legally blind by ~50% in nvAMD [9], by ~75% in DMO [10] and by ~50% in DR [11]. While this result is an enormous success and illustrates the value of targeting vessel leakage and angiogenesis to restore retinal function, it also highlights that there remains a huge number of patients who do not benefit from anti-VEGF therapies, benefit sub-optimally, or cease to respond [12]. This outcome can be ascribed to several factors: patients being underdiagnosed or diagnosed late, poor compliance to treatment, patient heterogeneity, progression to a different pathogenic stage of disease and to the intrinsic limits of VEGF-blockade strategy. Clinical trials have indeed shown that anti-VEGF therapy has reached an efficacy ceiling, whereby increasing the dose has no additional beneficial effect on visual acuity [13**–**15]. This could be due to compensatory signalling triggered by excessive VEGF-blockade such as that driven by the angiogenic factor angiopoietin-2. In fact, Faricimab, a bispecific antibody targeting both VEGF and angiopoietin-2, has given promising results in phase III trials for DMO and nvAMD [16] though it is not yet clear whether Faricimab is effective in those patients who fail to respond or respond poorly to the anti-VEGFs.

Concern regarding VEGF neutralisation also derives from its role as a vascular survival factor. Although the safety record of intraocular delivery is excellent, prolonged anti-VEGF treatment in cancer, for example, is linked to several adverse effects associated with vascular dysfunction: hypertension, microangiopathy, cardiac ischaemia, thromboembolic events and gastrointestinal bleeding [17]. VEGF is also thought to play an important role in maintaining the specialised fenestrated phenotype of the choriocapillaris and loss of fenestrations has been observed following VEGF-blockade possibly resulting in compromised function of this vascular bed [18]. Indeed, as the vascular response to VEGF may be finely tuned by differential signalling to the apical and basal side of the endothelium [19], disturbance of this delicate balance may impact on normal function. Moreover, although VEGF tropism initially seemed specific to the vasculature, hence the name, we now know that other retinal cell types, including RPE cells, Müller cells and astrocytes, express VEGF receptors thus raising concern regarding the safety of an indiscriminate long-term blockade of this factor [20**–**22]. Additionally, VEGF blockade does not address the inflammatory and fibrotic components that are usually present in retinal diseases and that are often the main cause of visual impairment. In summary, there is an urgent need to identify new pathogenic targets and develop novel therapeutics for retinopathies to use either as monotherapy or in conjunction with existing standard of care.

In pursuit of the above objective, leucine-rich α-2 glycoprotein 1 (LRG1) has been identified over the preceding decade as a vasculopathic factor that contributes to the pathogenesis of a variety of diseases including cancer, nvAMD, DR and kidney disease [23**–**30]. Under normal conditions this secreted protein is primarily produced by hepatocytes and granulocytes, but in both acute and chronic pathological states is often found increased in plasma. Indeed, in cancer patients a high plasma concentration often correlates with poor prognosis. The increasing evidence that LRG1 is pathogenic is driving a growth in research aimed at deciphering its physiological function, pathogenic role and whether it can be used as a diagnostic biomarker or therapeutic target. Despite gaps in our understanding of its biology, it is now clear that one of its main modes of action is in modulating transforming growth-factor β (TGFβ) signalling and as such it plays pivotal roles in both neovascularisation [23] and fibrosis [31]. It is also involved in immune responses and has been described as a potential acute-phase protein in that its hepatic expression is enhanced by systemic inflammation [32]. In this review, we first compile the evidence for the multifaceted roles of LRG1 as a vasculopathic, pro-fibrotic and immunomodulatory factor, and consider how these pleiotropic roles may contribute to eye diseases. We then describe the pre-clinical evidence for a pathogenic role of LRG1 in DR and nvAMD, and touch upon other ocular pathologies. Finally, we will highlight the potential of therapeutic targeting LRG1 for the treatment of eye disease in which there is a vascular component.

## LRG1 structure and physiological expression

**Fig. 1:**
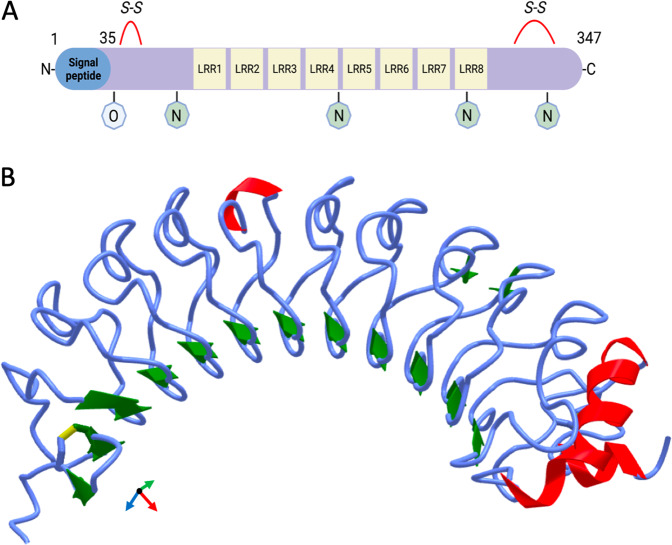
Structure of human Leucine-rich α-2 glycoprotein 1 (LRG1). **A** LRG1 is a 312 aa protein containing eight leucine-rich repeats (LRR), evolutionarily associated with protein–protein interaction, four N-linked and one O-linked glycosylation sites and two disulphide bonds. Following processing of the N-terminal signal peptide, the mature 50 kDa form of LRG1 is released into the extracellular space. **B** ALPHAFOLD2 prediction of LRG1 structure by deep learning algorithm [147]. β-sheet in green, helix in red.

LRG1 was first isolated from human serum by Haupt and Baudner in 1977 [33]. It is a member of the leucine-rich repeat family and contains 8 leucine-rich repeats, at least 4 N-linked glycosylation sites and 2 disulphide bonds (Fig. 1). Following processing of the N-terminal signal peptide, the mature 50 kDa protein is released into the extracellular space. Under homoeostatic conditions, LRG1 is almost exclusively synthesised by the liver and granulocytes [34] and is found in plasma at a concentration of 10–50 µg/ml [35]. Since its discovery, much has been unravelled about the role of LRG1 in pathology but little is known about its physiological function. Cummings et al. serendipitously identified a strong interaction between plasma LRG1 and cytochrome c (CytC) [36] an interaction which has been recently described to happen also intracellularly [37]. CytC is a small soluble electron carrier hemeprotein involved in the respiratory chain and, as such, is localised to the inner membrane of the mitochondria. When mitochondria are damaged, CytC is released into the cytosol where it initiates apoptosis. As a result of cell death or necrosis, CytC is then released into the extracellular space, where it can act as a danger-associated molecular pattern signal and trigger systemic inflammation. For example, intra-articular injection of CytC in mice induces substantial immune recruitment and symptoms like those of rheumatoid arthritis [38]. A plausible hypothesis is therefore that low levels of circulating LRG1 could be constitutively released to counter-act the deleterious effects of released CytC and avoid a systemic activation of the immune system in the absence of a committed inflammatory reaction.

In support of this idea, it was later demonstrated that the presence of LRG1 in culture medium protects lymphocytes from the pro-apoptotic effects of exogenous CytC [39], implying that LRG1 may exert a homoeostatic role in regulating lymphocyte number. This hypothesis has further implications since several cell types are susceptible to CytC toxic effects, including neurons [40], meaning that LRG1 could have a broader homoeostatic role. However, such a role remains unclear as *Lrg1*-deficient mice develop normally and have no overt phenotype suggesting that developmental or homoeostatic roles are not essential, or that they can be compensated for by other pathways. This is important knowledge from a therapeutic standpoint for it implies that, in contrast to VEGF, blockade of LRG1 should have little or no adverse effects on healthy tissues. This must be considered, however, with the caveat that these studies have all been conducted on specific pathogen free laboratory animals.

## LRG1 pathogenic mechanisms

### LRG1 as a promoter of vascular dysfunction and pathological angiogenesis

The presence of a dense neural layer makes the retina one of the most metabolically active tissues in the body and, as such, it is particularly reliant on the retinal and choroidal vasculature for appropriate provision of nutrients and oxygen. Vascular homoeostasis is the fundamental process that maintains the endothelium in a healthy state. It is an active process, centred around upkeep of EC quiescence through autocrine and paracrine signalling, physical contact with perivascular cells, sensing of the extracellular matrix (ECM) composition, and mechanically, through a constant physiological blood sheer stress. It is unsurprising then that a pathological perturbation of this finely tuned equilibrium triggers vascular instability that may lead to leakage, hypoxia, and growth of abnormal vessels. In the early stages of DR, for example, altered signalling triggered by hyperglycaemia stimulates detachment of pericytes from capillaries depriving ECs of quiescence signals and results in aneurism, haemorrhage and microangiopathy; in the more advanced stage of DR, poor perfusion, hypoxia and loss of homoeostatic signalling from pericytes and vessel drop-out will trigger angiogenic sprouting [41]. In nvAMD increased local expression of VEGF, secondary to hypoxia, promotes hyper-permeability, and prolonged exposure to this growth-factor stimulates the formation of new abnormal capillaries which impair central vision if affecting the macular region [42]. Vascular dysfunction is also a feature of other conditions such as macular telangiectasia, Coat’s disease and radiation retinopathy, while pathological angiogenesis is relevant to nvAMD, Coat’s disease [43], retinal vein occlusion [44] and choroidal haemangiomas [45]. Choroidal neovascular membranes can develop as a consequence of high myopia, ocular histoplasmosis, punctate inner choroiditis, multifocal choroiditis and central serous chorioretinopathy, as well as in macular telangiectasia [46]. While pathological angiogenesis shares some of the characteristics of physiological angiogenesis, it differs dramatically in that the resulting vessels are frequently dysfunctional being more tortuous, permeable and less-well perfused. The reasons for this remain poorly understood and are a scientific conundrum that has yet to be fully explained. Nevertheless, it points to the presence of disruptive factors in disease that are not present during developmental or physiological angiogenesis. Pathological vascular dysfunction has been well documented in cancer where it is associated with hypoxia and inefficient penetration of therapeutic agents [47], but it is also a feature of the ocular vascular diseases described above, and the potential contribution of LRG1 to this is discussed in greater detail below.

**Fig. 2:**
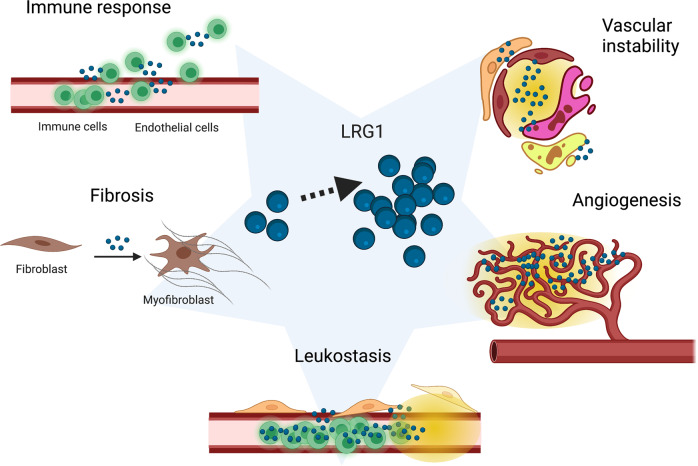
Pathological roles of LRG1. Increased levels of LRG1 are often reported in disease and several pathogenic mechanisms involving LRG1 have been proposed. LRG1 can play a part in the immune response by modulating lymphocyte number, granulopoiesis and neutrophil function and possibly by regulating TGFβ-mediated EC-leukocyte interactions. Many cell types like fibroblasts, epithelial cells, endothelial cells and pericytes can undergo trans-differentiation to myofibroblasts and contribute to fibrosis, and LRG1 has been implicated in the conversion of fibroblasts into fibrogenic myofibroblasts in a model of lung fibrosis. Leukostasis is a feature of DR in which neutrophils adhere to the non-perfused capillaries; LRG1 might be released by adherent neutrophils and mediate the EC damage which is associated with leukostasis. LRG1 upregulation during pathological angiogenesis is required for the TGFβ-induced angiogenic switch of ECs. Vascular instability/dysfunction follows altered physical and chemical interactions between ECs and pericytes; the TGFβ family of ligands and receptors plays an essential role in vessel maturation and homoeostasis by regulating these interactions, LRG1 has been shown to impact TGFβ signalling on both ECs and pericytes. Created with BioRender.com.

LRG1 is one of the most recently identified promoters of vascular dysfunction and pathological angiogenesis (Fig. 2), being up-regulated in laser-induced choroidal neovascularisation (CNV) and oxygen-induced retinopathy (OIR) [23]. Enriched in these and several other pre-clinical models of vascular remodelling (reviewed in the following sections), LRG1 has been shown to modulate TGFβ signalling in ECs, switching their phenotype from a quiescent to an active angiogenic state [23]. Arguably one of the most complex signalling networks, not only for the number of family members, but also for its high degree of cell, stage- and context-specific signalling outcomes, TGFβ plays a central role in vascular homoeostasis, exemplified by inherited diseases like Marfan and Loeys–Dietz syndromes where excessive TGFβ signalling leads to severe vasculopathy [48].

**Fig. 3:**
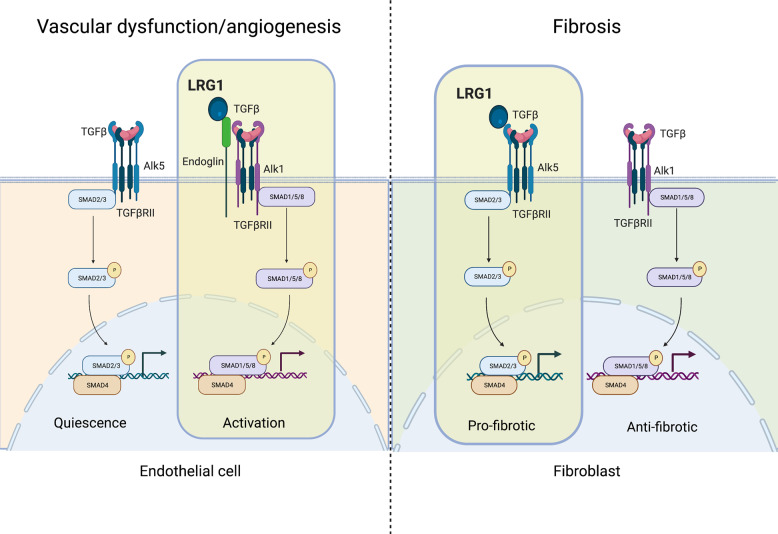
Context-dependent modulation of TGFβ pathway by LRG1. LRG1 can direct TGFβ signalling towards a specific pathway depending on the cell type and the context. During pathological angiogenesis/vascular dysfunction (left), LRG1 binds to Endoglin and switches the EC phenotype towards a pro-angiogenic state in an ALK1/SMAD-1,-5,-8-dependent fashion [23]. During fibrosis (right), LRG1 was shown to promote differentiation of fibroblasts into matrix-producing myofibroblasts by favouring the ALK5/SMAD-2,-3 pathway in an Endoglin-independent way [31]. Created with BioRender.com.

LRG1 binds to the TGFβRII accessory receptor endoglin (ENG) and in conjunction with TGFβ it promotes phosphorylation of SMAD-1 and -5 through ALK1. This leads to an increase in EC proliferation, migration and tubulogenesis and to new blood vessel formation in both the ex vivo metatarsal model and in vivo CNV model, effects which can be reversed by genetic deletion of ENG [23]. Interestingly, in vitro analysis revealed an interaction also between LRG1 and ALK5, which is out-competed by ENG and ALK1, suggesting that LRG1 could have different effects on the TGFβ pathway depending on the cell-type-specific spectrum of TGFβ receptors expressed [23] (Fig. 3). Interestingly, ENG is required for TGFβ-induced vasodilation through regulation of endothelial nitric oxide synthase (eNOS) abundance and NO synthesis [49]. Notably, LRG1 plasma levels correlate with arterial stiffness and reduced vasodilation in patients with type 2 diabetes, which could therefore perhaps be explained by LRG1 tuning of EC TGFβ signalling [50].

Recently, pericytes have also been added to the list of cell types amenable to LRG1 effects. In a model of Lewis lung carcinoma (LLC) LRG1 appears to promote an increase in NG2^+^ perivascular cells which are proposed to facilitate metastasis [51]. Interestingly, anti-LRG1 antibody treatment showed efficacy in prolonging overall survival of mice injected with LLC cells [51] which is amongst the tumour models that show a poor response to VEGF blockade [52]. This is, of course, of great relevance to eye diseases, where anti-VEGF therapy is the standard of care, and suggests that LRG1 targeting is an avenue that warrants exploration. Unpublished data from our lab are also suggestive of a direct effect of LRG1 on pericytes, both in the context of early-stage diabetic retinopathy (DR) and in the context of pathological neovascularisation. In particular, we observed that LRG1 negatively regulates pericyte vessel coverage, a prerequisite of vessel maturation and vascular homoeostasis. Moreover, this observation that LRG1 destabilises vessels may explain why its deletion or inhibition results in reduced neovascularisation. Thus, a destabilised vessel will be more prone to sprouting angiogenesis and so stabilisation will reduce vessel growth.

**Fig. 4:**
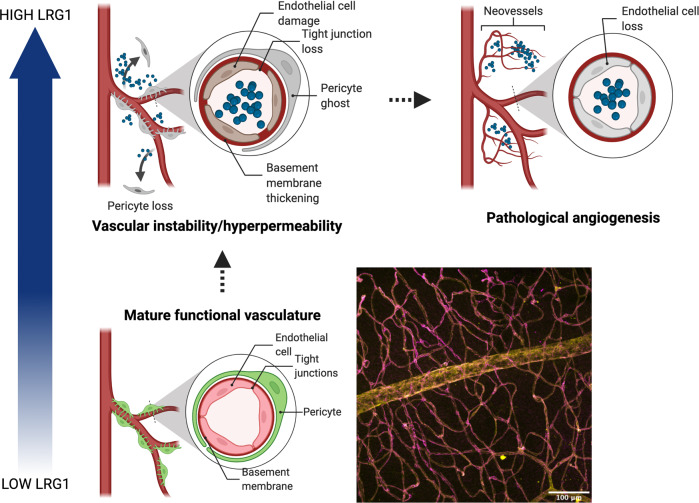
LRG1 vasculopathic effects. A mature functional vessel is characterised by extensive pericyte coverage of the endothelium, and tight junctions between adjacent ECs to maintain barrier function. Confocal 3D reconstruction of murine retina showing high pericyte (yellow) coverage of ECs (magenta). In homoeostatic conditions, LRG1 is found at low levels in the circulation and is undetectable in the retinal tissue. In pathological scenarios, such as nvAMD and DR, LRG1 is upregulated both locally and systemically and contributes to vascular instability by altering pericyte-EC interactions and by directly promoting EC activation. These pathological alterations can result in loss of vessel homoeostatic function (i.e. barrier properties) and ultimately lead to the growth of new pathological vessels. Created with BioRender.com.

In summary, there are two mechanisms through which LRG1 can compromise vascular stability: by directly activating the TGFβ-dependent angiogenic switch on ECs and by hindering pericyte coverage of the endothelium and therefore suppressing vessel quiescence (a prerequisite for tip cell formation) (Fig. 4). Accordingly, the evidence now points to LRG1 being a key player in driving vascular dysfunction in disease including those vascular complications observed in the eye.

### LRG1 pro-fibrotic role

Fibrosis refers to the excessive deposition of ECM proteins that accompanies many pathological conditions. While in some instances, such as following acute tissue injury, a fibrogenic wound-healing programme is required to restore basic tissue integrity, in most cases this response is prolonged or dysregulated with irreversible deleterious effects on tissue/organ function. Two major cell types drive fibrosis in eye disease, namely fibroblasts and glial cells (Müller cells, astrocytes and microglia). Fibrosis-derived scarring of the eye has serious consequences as it impacts vision both mechanically, by opacifying the visual axis, and biologically, by damaging tissue homoeostasis and cell function [53]. For example, fibrosis in the cornea leads to opacification and this is often observed following viral infection [54]. It is well established now that pathological vascularisation is a trigger of gliosis, and this is particularly evident in the advanced stage of DR which is characterised by epiretinal fibrous membrane. Here, new blood vessel formation is accompanied by glial cell activation and proliferation and, as neo-vessels penetrate the vitreous, they contract leading to retinal detachment [55]. A similar process is observed sub-retinally in nvAMD, where abnormal blood vessels first proliferate under the Bruch’s membrane and the RPE and then invade the sub-retinal space, leading to haemorrhages, leakage, serous retinal detachment, and scarring [56]. Angiogenesis-induced gliosis is also relevant to retinopathy of prematurity [57, 58] and can be a complication of retinal detachment surgery [59]. Excessive production of ECM components can also happen independently of angiogenesis; in the early stages of DR, for example, thickening of the basal lamina surrounding capillaries is thought to be the initiating step that precedes the loss of retinal ECs and pericytes [55, 60].

Fibrosis/gliosis in the eye and other organs shares common features, one of which is the dominant role of TGFβ. This pleiotropic cytokine is the master regulator of matrix deposition and a potent inducer of cell conversion to myofibroblasts. These are actin-myosin-rich cells, tightly connected to the ECM, extremely contractile and characterised by high production of matrix proteins. Fibroblasts are the cell type most prone to transdifferentiate into myofibroblasts, but not the only one. There is some evidence of vascular cell conversion. ECs, for example, can undergo TGFβ-induced endothelial-to-mesenchymal transition (EndMT) and contribute to matrix deposition in a variety of experimental models [61]. Pericytes can also become activated and acquire a myofibroblast-like phenotype in vitro, although little is known about the implications of this process in in vivo [62, 63]. In proliferative vitreoretinopathy, which is often secondary to rhegmatogenous retinal detachment or major ocular trauma and surgery, a crucial cellular component of the fibrotic lesion is RPE cell trans-differentiation into myofibroblasts via epithelial to mesenchymal transition [64]. In addition to the effects described above, TGFβ can also exert a pro-fibrotic function by acting directly on tissue-resident macrophages, increasing their production of pro-fibrotic cytokines which in turn activate fibroblasts. TGFβ can also act as a potent chemotactic factor for monocytes, and can therefore enhance their recruitment to the fibrotic lesion where they can then differentiate into fibrosis-conducive macrophages [65].

Recent work revealed a strong immunostaining of LRG1 in the neovascular lesions of treatment-naïve nvAMD patients, particularly concomitant with myofibroblasts and ECs [30]. Interestingly, an involvement of LRG1 in fibrosis does not appear to be organ-specific as it is also found upregulated in infiltrated immune cells, as well as bronchial epithelial cells, in a bleomycin-induced murine model of lung fibrosis. Consistent with the previously described role of LRG1 as a modulator of TGFβ signalling [23], *Lrg1*-deficient mice are protected from developing fibrotic lesions following bleomycin injection. In agreement with involvement of the TGFβ/SMAD pathway, *Lrg1*-deficient lungs presented a reduced pSMAD2 signal. In vitro analysis further revealed that fibroblasts are responsive to LRG1 and exhibit enhanced SMAD-2 signalling when LRG1 and TGFβ are present in the medium, resulting in a pro-fibrotic gene signature [31] (Fig. 3). Interestingly, this mechanism appears to be context-dependent. In the heart, for example, a study using pressure overload as a model of cardiac fibrosis showed that *Lrg1*-deficient mice present with exacerbated fibrotic cardiac remodelling [66]. Here, cardiac fibroblasts derived from *Lrg1* knock-out mice were shown to be more migratory, contractile and express higher levels of fibrotic proteins due to increased TGFβ/SMAD-2 signalling. LRG1 therefore appears to inhibit the TGFβ pro-fibrotic effect in this context and the authors propose a model whereby LRG1 constitutively expressed at low level out-competes TGFβ for TGFβRII binding and fibrotic genes are thus repressed. During cardiac fibrosis, however, LRG1 is reduced and TGFβ pro-fibrotic signalling prevails. Interestingly, in this setting, an additional level of complexity is added by the Silencing Mediator of Retinoid and Thyroid (SMRT) hormone receptor which, induced upon pressure overload, represses *Lrg1* transcription providing a self-amplification circuit for TGFβ pro-fibrotic signalling [66]. These pre-clinical results in different organs could be indicative of a role of LRG1 in fibrosis of other tissues, including the eye (Fig. 2). Interestingly, IL-6, which is found increased in the vitreous aspirates from both PDR [67] and nvAMD [68] patients, has been shown to have pro-fibrotic activity in a pre-clinical model of sub-retinal fibrosis [69]. We have evidence, from unpublished work in our lab, that IL-6 upregulates *LRG1* expression in ECs. This could imply a feed-forward mechanism between angiogenesis and fibrosis, in which IL-6 and LRG1 are key components and potential targets.

### Immunomodulatory roles of LRG1

Traditionally, the eye is considered a site of immune privilege, owing to the mechanical barrier provided by the blood–retinal barrier and an immunosuppressive environment. This evolutionary adaptation is thought to have arisen to protect vision from the damaging effects of swelling and hyperthermia which accompany the flogistic reaction. Thus unsurprisingly, excessive or chronic ocular inflammatory responses are pathogenic and play a central role in some of the most common eye conditions. For instance, chronic low-grade inflammation or “para-inflammation”, associated with age-related accumulation of oxidised lipoproteins and free radicals, has been recently described as a potential reason for RPE and photoreceptor loss in the elderly and a contributing factor to AMD [70, 71]. Moreover, in AMD, RPE cell injury releases inflammatory factors that recruit dendritic cells from the choroid, which in turn amplify the inflammatory reaction by forming immune complexes and triggering the complement system. Overt activation of the complement system, elements of which are found in the drusen of AMD patients [72], appears to have a critical role in AMD pathogenesis, a notion strongly supported by a number of genetic studies where variants in genes of the complement system (e.g. complement factor H) show strong association with the disease [73–75]. Inflammation is also a pathological component of DR, whereby chronic hyperglycaemia promotes EC activation and local production of a pro-inflammatory milieu which includes IL-6, IL-8, TNFα, VEGF and MCP-1 [76]. Increased levels of chemoattractant and of adhesion molecules on the luminal side of blood vessels, such as ICAM-1 and VCAM-1, promote leukocyte adhesion and trans-endothelial migration [77]. Leukocyte transmigration triggers an increase in permeability, which is also sustained by a direct effect of inflammatory mediators on EC junctional stability and can result in the vascular leakage observed in DMO [78]. VEGF is one of the most potent vascular permeability factors and its role in junctional remodelling is well documented in retinopathy [79]. Moreover, the overt endothelial adhesiveness coupled with narrowing of the capillaries promotes leukostasis, which results in capillary occlusion and also EC and pericyte apoptosis due to lack of blood flow and release of cytotoxic by-products [80, 81]. Therapeutically targeting the inflammatory response in the eye to restore tissue homoeostasis has vast potential and the use of corticosteroids has been a standard approach for a number of ocular diseases [82].

The *Lrg1* promoter contains STAT and NFκB responsive elements, so it is unsurprising that its expression often correlates with inflammatory responses. Expression by hepatic cells is upregulated by acute-phase mediators such as IL-6 and LPS, leading to elevation of LRG1 in the systemic circulation [32]. LRG1 has also been proposed as a biomarker for rheumatoid arthritis [83], lupus erythematosus [84], asthma [85], ulcerative colitis [86], psoriasis [87], lupus nephritis [88] and Still’s disease [89]. As discussed in section 2, LRG1 could influence lymphocyte populations by protecting them from the cytotoxic actions of circulating CytC [39]. In addition, in a murine model of collagen-induced arthritis (CIA), *Lrg1*-deficient mice showed reduced disease burden. The authors further showed that in vitro LRG1 enhances expression of the IL-6 receptor on naïve T cells through the TGFβ/Smad2 axis, enabling differentiation to disease-conducive Th17 cells. Interestingly, this study also reported an enhanced phosphorylation of p38 by LRG1, consistent with the survival effect of LRG1 on lymphocytes previously reported [39]. This pathway should be relevant in vivo, where *Lrg1*-deficient mice have a reduced T helper compartment [83].

In experimental auto-immune uvoretinitis (EAU), an animal model of uveitis with clinical-pathological features remarkably similar to human disease [90], Th17 (and Th1) T helper cells are important inducers of the disease, for their secretion of cytokines like IL-17 and IFN-γ that allow neutrophil and macrophage tissue infiltration [91, 92]. It would be therefore interesting to explore whether LRG1 modulates Th17 differentiation in this context. Interestingly, there is evidence of LRG1 being a marker of high-endothelial venules, a subtype of ECs specialised for the recruitment of lymphocytes from blood to the tissue interstitium [93, 94], although the functional significance of this finding is still unclear. Nevertheless, knowing that TGFβ negatively regulates EC-lymphocyte adhesion [95, 96], one could speculate that the presence of LRG1 at sites of tissue extravasation could finely tune TGFβ signalling and as such influence initiation and termination of lymphocyte migration across the vessel wall. LRG1 can also potentially modulate neutrophil function. Its expression is switched on during G-CSF-induced granulopoiesis and it can be detected in granulocytes throughout their differentiation into neutrophils [97]. Despite generally being considered a secreted protein, LRG1 appears to be contained in myeloperoxidase-rich granules and, interestingly, the *LRG1* gene locus is proximal to several genes encoding neutrophil granule enzymes (19p13.3). Moreover, overexpression of LRG1 in the murine myeloid cell line 32Dcl3 led to increased expression of the neutrophil marker CD11b and accelerated differentiation into neutrophils, possibly through augmentation of STAT3 phosphorylation [98]. More recently, LRG1 was shown to increase adhesion of the neutrophil cell line dHL-60 onto EC monolayers in vitro, possibly through upregulation of the adhesion molecule L-Selectin on neutrophils. In the context of diabetic wound healing, LRG1 appears to promote neutrophil extracellular trap formation (NETosis) in a TGFβ/SMAD5/AKT -dependent manner [99].

In summary, targeting LRG1 could affect the inflammatory component of some eye diseases in multiple ways: through direct effects on the vasculature, via modulation of leukocyte recruitment and leukocyte differentiation/number (Fig. 2).

## Clinical and pre-clinical evidence of a role for LRG1 in eye diseases

### LRG1 in diabetic retinopathy

DR is the leading cause of sight loss in the working age population of industrialised countries and the fifth leading cause worldwide. It is the most common complication of diabetes and its occurrence is strongly associated with the duration and severity of hyperglycaemia (usually more than 20 years), hypertension, hyperlipidaemia and smoking. DR is initially characterised by a non-proliferative stage (NPDR) during which the first microvascular abnormalities accumulate, such as microaneurysms and retinal haemorrhages which are likely secondary to basal lamina thickening and pericyte loss. These initial signs are important for the diagnosis of DR and may help to assess the risk for progression to sight-threatening DR. Leaky microaneurysms and pericyte-depleted capillaries are also a source of hard exudates (lipid and lipoprotein deposits), which are often observed in the outer layer of the retina. Concomitantly, poor perfusion and pericyte loss can give rise to occluded and de-endothelialised capillaries, that appear as dark areas in fluorescein angiograms. Insufficient perfusion of acellular capillaries also triggers nerve fibre ischaemia and axonal swelling, clinically identified as “cotton wool spots”. The disease can, in some cases, further progress into a proliferative stage (PDR), where local hypoxia drives upregulation of pro-angiogenic factors and subsequent formation of new abnormal blood vessels which can haemorrhage and impair vision. DMO is a complication of DR which can develop at any stage of the disease and is characterised by swelling of the macular area secondary to fluid extravasation. The molecular framework of DR is extremely complex with several pathways playing a role in the disease, including insulin signalling, hypoxia, inflammation, lipid metabolism, neurogenesis and VEGF-induced permeability and angiogenesis [100]. Targeting of VEGF has revolutionised the treatment of PDR and DMO but, regrettably, is only effective in arresting or slowing down disease progression in approximately half of the patients and often the efficacy is short lived [1]. This suggests that other factors promote vascular dysfunction in DR and/or could compensate for VEGF once it is therapeutically blocked.

**Table 1:** Proteomic analysis of therapeutic targets Leucine-rich α-2 glycoprotein 1 (LRG1), vascular endothelial growth factor (VEGF) and Angiopoietin-2 (ANG2) in diabetic retinopathy (DR), neovascular age-related macular degeneration (nvAMD) and retinopathy of prematurity (ROP).

Source tissue	Disease	LRG1 increased vs. control	VEGF increased vs. control	ANG2 increased vs. control	Author, Ref.
Vitreous	nvAMD	Yes	No	No	Nobl et al. [121]
Aqueous	AMD	Yes	No	No	Qu et al. [122]
*Post-mortem* retina	Dry and nvAMD	Yes	No	No	Yuan et al. [123]
Vitreous	PDR	Yes*	No	No	Kim et al. [106]
Vitreous	Non-PDR and PDR	Yes*	No	No	Gao et al. [104]
Vitreous	PDR	Yes	Yes	No	Zou et al. [107]
Vitreous	PDR	ND	Yes	ND	Mitamura et al. [148]
Vitreous	PDR	ND	Yes	Yes	Watanabe et al. [149]
Vitreous	Non-PDR and PDR	Yes	Yes	ND	Chen et al. [105]
Vitreous	NPDR with DMO	ND	ND	Yes	Patel et al. [150]
Cord blood	ROP	Yes	No	No	Zasada et al. IOVS 2018 [151]

A summary of studies that have analysed the proteome of ocular disease samples highlighting whether LRG1, VEGF and ANG2 are found elevated in disease versus controls.

*LRG1 precursor.

Cross-sectional studies reported LRG1 levels to be increased in diabetic patients’ plasma [50, 101] and urine [102] and has been proposed as a possible biomarker for DR [103]. Notably, proteomic analyses of vitreous humour from DR patients have revealed LRG1 as an upregulated hit in numerous independent studies [23, 104–107] (Table 1). Whether vitreous LRG1 is partly serum-derived through the leaky vasculature or is locally produced, remains to be established although animal studies would indicate local contribution is at least a contributing factor. Longitudinal studies will also be required to elucidate whether an increase in LRG1 is causative to DR onset and/or progression or whether it is a consequence of the diabetic state. Animal studies, however, would argue for a pathogenic role of LRG1 at least in the neovascular end stage of the disease. In fact, pre-clinical studies using OIR as a model of retinal neovascularisation revealed that the *Lrg1* retinal transcript is upregulated during the pathological angiogenic phase and that lack of LRG1 protects mice from formation of neovascular tufts [23]. TGFβ is known to promote DR progression at the late stage of the disease [108] and the pro-angiogenic role of LRG1 is mediated by switching TGFβ signalling on ECs towards the angiogenic pathway [23]. While these investigations establish a novel mechanism of pathological neovascularisation likely to be relevant in human disease, it is still largely unknown what drives the initial microvascular dysfunction prior to angiogenesis. The underlying biology is certainly complex, considering for example that TGFβ alone, which is pro-angiogenic towards the end stage of DR, has a protective homoeostatic role at the beginning, as demonstrated by a conditional knock-out of TGFβRII in ocular tissue which results in DR-like retinopathy [109]. Given that LRG1 expression is switched on from the early onset of experimental DR, it would be interesting to evaluate the phenotype of LRG1-deficient mice at the early stages of the disease to assess whether LRG1 plays a role then, possibly priming ECs towards dysfunction in a TGFβ-dependent fashion. Moreover, other cell types in the retina express receptors for TGFβ possibly increasing the spectrum of cells susceptible to an excess of LRG1 in the retinal microenvironment. Pericyte-EC interactions, for instance, are highly dependent on TGFβ, which is expressed by both cell types, and regulates their proliferation and differentiation. Interestingly, TGFβ activation from the latent form requires contact between EC and pericytes [110], so in the context of DR, it would be valuable to evaluate the TGFβ pathway following pericyte detachment from ECs and how this could be modified by LRG1. Our data from cancer studies indicate that LRG1 affects pericyte investment of vessels [111] and would suggest, therefore, that in diabetes LRG1 may contribute to both early (development of vascular instability) and late (neovascularisation) events in its pathogenesis.

DR is also characterised by retinal inflammation, and increased levels of IL1β, TNFα, ICAM-1 and angiotensin II are often detected in the vitreous of diabetic patients [112, 113]. These factors contribute to EC activation and leukostasis, and adherence of leukocytes onto the vessel wall, which ultimately leads to EC damage and hyper-permeability.

Gene deletion studies in mice have provided strong evidence for a central role of the transcription factor NFκB in DR pathogenesis [114] and, interestingly, the *LRG1* promoter contains NFκB responsive elements which could explain the local upregulation of LRG1 during the early stages of DR. IL-6, a pro-inflammatory cytokine secreted by lymphocytes, monocytes, fibroblasts and ECs [115], is also found elevated in aqueous humour (AH) and vitreous of patients with DR [116–118]. Beside its pro-inflammatory effects, IL-6 can stimulate angiogenesis in tumour models with the new vessels characterised by decreased pericyte coverage, an effect which can be reverted by IL-6 blockade [119]. Similarly, unpublished data in our lab, describe a link between IL-6 and defective angiogenesis which is mediated by an IL-6-dependent upregulation of LRG1 in ECs. This nexus between IL-6, LRG1 expression and dysfunctional new blood vessels could be relevant in DR and requires further studies. Moreover, neutrophils can be detected adhered to the luminal surfaces of non-perfused capillaries in DR [120], and because LRG1 is highly expressed by granulocytes and able to affect their function, this raises questions as to whether adherent neutrophils could be a source of LRG1 in the diabetic retina, and what impact they might have on EC damage. Bone marrow transfers or conditional knock-out experiments could help understand the role of neutrophil-derived LRG1 in DR.

The evidence so far points to a potentially important role for LRG1 in DR where it may contribute to both the early and late proliferative phases of disease. Regarding the proliferative stage, where new dysfunctional vessels are pathogenic, much may also be learnt from studies in cancer. Here, an alternative therapeutic approach has gained traction, whereby strategies are being developed to encourage the vessel normalisation of existing and new vessels. The rationale for such an approach is that normal vessels reduce hypoxia, are less leaky and haemorrhagic and better able to deliver therapeutics. In the diabetic eye, new vessels are induced to counteract harmful hypoxia so ideally neovascularisation is required but such vessels need to be stable and functional. We propose, therefore that new vessel formation in the absence of LRG1 would be more likely to occur, as it does during development, in a physiological manner. Restoring pericyte coverage of vessels, re-establishing EC junctional stability, impeding leukocyte EC-adhesion, and generally re-establishing a quiescent endothelial cell state should therefore remain a critical objective for any new therapeutic targeting the vasculature (Fig. 4).

### LRG1 in age-related macular degeneration

Age-related macular degeneration is the leading cause of blindness in the elderly of industrialised countries and, with longevity increasing, its incidence is bound to rise. Traditionally, AMD is classified into 2 sub-types: a more common, slow-progressing dry/non-exudative form (accounting for ∼85% of patients) and a less common but more rapid progression wet/neovascular/exudative form. Both sub-types can be classified according to the severity of the lesions as early, intermediate or advanced. In the advanced stage of dry AMD, known as geographic atrophy, degeneration of the RPE, retina and the choriocapillaris leads to visual impairment. The precise aetiology of AMD is not known but it is clearly a multi-factorial disease, the complexity of which is reflected by its association with an increasing number of genetic variants. The hallmark features of the early stages of dry AMD are sub-RPE deposits or drusen, RPE abnormalities, hyperpigmentation and choriocapillaris loss. These changes are due to altered pathways including oxidative stress, cell senescence, mitochondrial dysfunction and inflammation. Once the RPE and photoreceptors are damaged, the loss of visual function is irreversible. In nvAMD, RPE damage, local hypoxia and other insults drive new vessel growth from the choroid into the macula. These neo-vessels, immature and leaky, contribute to fluid accumulation, haemorrhage and fibrosis which irreversibly disrupts central vision. While there is no treatment for dry AMD (although clinical trials with cell-based therapies and complement modulators are showing promise), the neovascular form can be treated with monthly or less frequent IVT injections of anti-VEGF pathway agents, since VEGF is one of the main drivers of permeability and angiogenesis in the disease. The advent of anti-VEGF drugs marked a breakthrough in the treatment of nvAMD, but their efficacy is not absolute, may be short lived, and persistent blockade of VEGF-induced survival signals could negatively impact ocular tissues.

Proteomic analyses identified LRG1 as an enriched component of the vitreous humour of patients with CNV and in both vitreous and Bruch’s membrane biopsy of dry AMD patients [121–123] (Table 1). Pre-clinical evidence demonstrates that LRG1 is barely detectable in a healthy retina and it is only in pathological scenarios that its expression increases significantly. For example, in a murine model of CNV, where new blood vessel growth from the choroid into the retina is triggered by laser injury to Bruch’s membrane, LRG1 is upregulated by 4-fold [23]. Interestingly, genetic depletion of *Lrg1* reduces both the angiogenic and the permeability responses in this model, an effect which can also be replicated by IVT injection of anti-LRG1 blocking antibodies. As is the case in OIR, the proposed mechanism by which LRG1 contributes to pathological neovascularisation is switching of the TGFβ signalling cascade towards the pro-angiogenic ALK1/SMAD-1,-5,-8 pathway on ECs. Of clinical relevance, the anti-angiogenic effect elicited by anti-LRG1 antibodies is of similar magnitude to that achieved by antibody blockade of VEGF/PLGF and, importantly, combinatorial treatment shows synergistic effects, suggestive of independent pathways at play [23].

Myofibroblasts are the principal cellular constituent of the fibrous membrane which generates secondary to sub-retinal fibrosis in CNV. These matrix-producing mesenchymal cells are thought to be generated, at least in part, from RPE cells by the process of EMT [56]. Interestingly, a recent in vitro study revealed that LRG1 is produced by RPE cells undergoing TGFβ-induced trans-differentiation, and knocking-down *Lrg1* abolishes this process. Moreover, NADPH oxidase 4 (NOX4), previously shown to participate in TGFβ-induced EndMT, appears to be regulated by the levels of LRG1 [124]. Recent published work corroborates the notion of both myofibroblasts and ECs being cellular sources of LRG1 in the neovascular lesions of treatment-naïve nvAMD patients [30]. Interestingly, in this study most patients treated with anti-VEGF agents exhibited reduced LRG1 expression associated with myofibroblasts and the vasculature, but some did not [30]. This raises the intriguing question of whether incomplete inhibition of LRG1 expression following VEGF-blockade, and presumably quiescence of the endothelium, makes a patient a non-responder. If so, then simultaneous blockade of VEGF and LRG1 would tackle a much larger group of patients. We now know that while anti-VEGF therapy usually improves visual function in nvAMD patients, sub-retinal fibrosis can develop in approximately half of patients and has been acknowledged as one of the principal causes of sight loss [125]. Thus, novel targets such as LRG1 that can not only tackle angiogenesis but also address fibrosis and potentially mitigate the fibrotic outcome of VEGF blockade are of particular interest.

### LRG1 in other ocular pathologies

i. Rhegmatogenous retinal detachment
  Retinal detachment (RD) is a serious condition which is caused when the neural retina separates from the RPE. There are three types of RD; Rhegmatogenous, Tractional and Exudative. Rhegmatogenous retinal detachment (RRD), is caused by vitreal fluid build-up in the sub-retinal space due to a defect in the retina and is the most common form of RD with an incidence of 6.3 to 17.9 per 100,000 population [126]. Novel proteomic studies have shed light on the molecular mechanisms of this disease by the detection of potential candidates that could play a role in the pathogenesis. In a recent study, vitreous samples collected from patients suffering from RRD (*n* = 127) were compared to vitreous samples collected from patients with Macular-Hole (*n* = 5), Pucker (*n* = 10) and PDR (*n* = 9) using SWATH-mass spectrometry. This study showed an upregulation of LRG1 (1.3 fold-change) in RRD compared to the other groups [127]. In another study, sub-retinal fluid (SRF) and vitreous were collected from patients with RRD and compared to samples from post-mortem eyes with no history of ocular disease using label-free quantification (LFQ). LRG1 was detected in both samples in RRD, with higher levels in the vitreous compared to SRF [128]. An additional study in 2018 revealed the presence of LRG1 in vitreous samples of patients with RRD (*n* = 4) and epiretinal membranes (MEM) (*n* = 4) by iTRAQ quantitative proteomics [129].
ii. Retinoblastoma
  Retinoblastoma (RB) is the most common paediatric intraocular cancer, which affects around 7500 children annually worldwide [130]. RB develops due to a mutation in the tumour suppressor gene RB1, which predisposes retinal cells to cancer [131]. The earliest form of treatment was enucleation, but modern medicine is moving towards a more conservative approach with gene therapy [132], chemotherapy, focal radiotherapy and laser therapies [133], and particular effort is being put into a search for new therapeutic targets. It was recently discovered that LRG1 may play an important role in tumour survival in RB [134]. Amer et al. [134] detected high expression of LRG1 by immunohistochemistry in RB samples from 34 patients. They also saw an increase in mRNA levels of LRG1 in RB tissue (*n* = 4) compared to controls. Another study also reported a significant increase in LRG1 mRNA and protein in RB tissue as compared to healthy tissue (*n* = 20) [135]. Importantly, they could also demonstrate that downregulation of LRG1 via silencing of nuclear paraspeckle assembly transcript 1 (NEAT1) can prevent tumour cell migration and invasion.
iii. Retinopathy of Prematurity
  Retinopathy of prematurity (ROP) is a potentially sight-threatening disease seen in preterm births. It is a result of oxygen insult to the immature retinal vasculature at birth which causes neovascularisation, RD or both [136]. One study showed a reduction in plasma LRG1 in the cord blood collected at delivery from new-borns that developed ROP compared to new-borns that did not. As this disorder affects the vessels of the retina, more molecular studies are needed to interrogate how and whether LRG1 plays a role in the progression of this disease.
iv. Age-related cataracts
  Cataract is a leading cause of blindness, leaving over 12 million people blind worldwide annually [137]. Cataracts are characterised by lens opacity thought to be caused by increased oxidative stress [138]. The AH is responsible for the transport of nutrients and removal of waste to and from the lens. Studies on the composition of the AH hence can give insights into pathogenesis. One of the early proteomic studies of AH using iTRAQ showed an increase in the concentration of LRG1 in patients with high myopia versus controls (*n* = 6). The authors also observed an increase in the abundance of LRG1 in patients after glaucoma surgery in comparison to controls (*n* = 6) [139]. More recently, a study using LC-MS/MS methodology detected high levels of LRG1 in 88 AH samples from patients undergoing cataract surgery.
v. Corneal neovascularisation (CNV)
  A lack of vasculature is responsible for corneal transparency, which is essential for visual acuity. Maintenance of this avascular state in the cornea is brought about by a balance of several pro-angiogenic and anti-angiogenic factors referred to as “angiogenic privilege” [140]. However, a number of pathological events such as infection, hypoxia due to alkali injury or contact lens use, inflammation, trauma, neoplasia can result in a loss of this protection and a sight-threatening invasion of new vessels into the cornea [141]. LRG1 plays a pivotal role in promoting corneal neovascularisation by upregulating the expression of VEGF and its receptors in a mouse model of CNV [142]. More recently, it was revealed that LRG1 can promote corneal fibrotic response and this is accompanied by neutrophil infiltration [143].
vi. Uveitis
  Uveitis is the inflammation of the uvea, which can be associated with auto-immune systemic diseases, infections or independent pathologies of the eye [144]. Experimental auto-immune uveitis mimics many aspects of the pathology and results in blood–retinal breakdown associated with retinal endothelial dysfunction. Recent transcriptomics of retinal ECs in EAU showed an upregulation of LRG1 during disease both at the transcript and protein level [145].

When considered individually, the weight of evidence supporting a pathological role for LRG1 in any of these diverse ocular pathologies may appear modest. But taken together, the consistent association of LRG1 with disease development and/or progression seems unlikely to be mere coincidence, particularly in light of what we know about LRG1 function. These correlative studies, plus the increasingly strong data around LRG1 involvement in AMD and diabetic eye disease, make a compelling case for assessment of therapeutic targeting of LRG1 in patients.

## Development of a therapeutic targeting LRG1

Our previous work [23] and the independent studies highlighted in this review prompted us to assess the therapeutic value of blocking LRG1 to inhibit pathological angiogenesis and stabilise the vasculature. Accordingly, we developed a humanised function-blocking antibody, named Magacizumab. We chose to develop an IgG4 isotype, since these are known to be less likely to evoke inflammatory responses compared to IgG1 or 2 isotypes, a characteristic particularly desirable in an intraocular injectable. IgG4 antibodies have a tendency to spontaneously undergo Fab arm exchange leading to the formation of hemibodies, however this was readily overcome by incorporation of the hinge-stabilising S-P^228^ mutation [146]. Magacizumab showed strong affinity to human LRG1, with an equilibrium dissociation constant (KD) of 2.81 × 10^−10^ M as measured by surface-plasmon resonance. Moreover, we could demonstrate its efficacy both as an anti-angiogenic agent and in inhibiting vascular leakage in a pre-clinical model of nvAMD [146]. These encouraging results led us to take this therapeutic a step forward and produce the derivative Fab fragment, named MagaFab, via papain digestion of the full-length antibody [146]. The advantages of using Fab fragments as therapeutic agents are: (i) the lack of Fc fragment, which may cause inflammatory reactions, (ii) their lower molecular weight which allows delivery at a higher molar dose and (iii) their adaptability to development as bispecifics. MagaFab showed slightly less affinity for human LRG1 compared to the parent antibody Magacizumab (KD Magacizumab = 2.81 × 10^−10^ M versus KD MagaFab = 4.4 × 10^−9^ M) but still achieved comparable inhibition of both pathological angiogenesis and reduced vascular leakage in a model of CNV [146]. These findings pave the way for the application of anti-LRG1 function-blocking antibodies for the treatment of a range of diseases characterised by vascular dysfunction and angiogenesis.

## Discussion

Retinopathies are sight-threatening pathologies that presently affect 2 million people in the UK. Where vascular complications occur the main therapeutic target of current drugs is the VEGF pathway, for its central role in permeability and angiogenesis. Despite anti-VEGF agents having revolutionised treatment of DR, DMO and nvAMD, they fail to deliver significant efficacy in all patients. Moreover, there are concerns regarding the long-term safety of this therapeutic strategy, given the role of VEGF as a survival factor in the retina. Furthermore, blockade of VEGF does not address fibrosis and inflammation which often occur concurrently with retinal disease and are strong contributors to an unfavourable prognosis. Targeting TGFβ could potentially tackle this problem, since this growth factor has pleiotropic roles in angiogenesis, inflammation and fibrosis, which are well documented in ocular pathologies. Unfortunately, despite this strong evidence, therapeutic targeting of TGF-β (or its receptors including ALK1 and ENG) is hampered by its multiple homoeostatic housekeeping roles, which would be suppressed by unselective TGFβ blockade with adverse consequences.

LRG1 is a secreted glycoprotein which is strongly upregulated in pathological settings where it promotes vascular destabilisation, inflammation and fibrosis. Crucially, unlike VEGF and TGFβ, LRG1 is neither required for development nor for homoeostatic physiological functions, a point corroborated by *Lrg1*-deficient mice not having an overt phenotype. Pathological levels of LRG1 on the other hand, frequently driven by ectopic overexpression of LRG1 at the sites of pathology as observed in some cancers, inflammatory conditions and eye disease, have strong biological activity which appears, at least in part, mediated by coercion of TGFβ signalling (Fig. 3). Targeting LRG1 could therefore indirectly hamper the pathogenic arm of TGFβ signalling while sparing its homoeostatic functions. Moreover, the timescale of LRG1 expression, especially in DR, would point to an early role for this molecule in the disease, when microvascular damage accumulates and no signs of neovascularisation are yet detectable. Normalising the vasculature at this stage by blocking LRG1 could potentially prove a more timely and effective treatment for DR patients, preventing subsequent conversion to DMO or PDR. There may also be benefits in dual targeting of LRG1 and VEGF since they represent distinct signalling pathways, either through combination therapy or the generation of a bispecific.

The encouraging pre-clinical results summarised in this review, led to the development of Magacizumab, a humanised/de-immunised anti-LRG1 monoclonal antibody and its Fab fragment (MagaFab). Preliminary experiments in murine models of ocular neovascularisation using these function-blocking antibodies confirmed inhibition of angiogenesis and vascular leakage, without any detectable toxicity or inflammatory response [146]. Fab fragments have the advantage of lacking potentially inflammatory Fc domains and, having a lower molecular weight, permit delivery at higher molar doses, which is particularly relevant in the context of ocular injections. Of note, smaller therapeutics and with simpler tertiary structures, such as Fab fragments, are advantageously more amenable to alternative delivery methods, such as gene delivery and slow-release formulations. There is a lot still to understand about LRG1 biology; the marked periodicity in leucine residues, which are sites designated to protein–protein interactions, would suggest the possibility of multiple interacting partners, of which perhaps only a few have been identified. Nonetheless, the current evidence from clinical and pre-clinical studies points toward a multifaceted pathogenic role for LRG1 in many diseases, including DR and nvAMD. Given that there is an urgent need for novel therapeutic strategies to address patient non-responders and loss of efficacy of anti-VEGF drugs and irreversible fibrosis in these pathologies, LRG1 certainly holds considerable promise as a candidate therapeutic target. Further research and clinical trials will increase our understanding of LRG1 pathophysiological roles and possibly pave the way to improved therapies for patients with eye disease.
