# Correction: LRG1 as a novel therapeutic target in eye disease

**DOI:** 10.1038/s41433-022-01988-6

**Published:** 2022-02-28

**Authors:** Giulia De Rossi, Marlene E. Da Vitoria Lobo, John Greenwood, Stephen E. Moss

**Affiliations:** Present Address: Institute of Ophthalmology, University College London, 11-43 Bath Street, London, EC1V 9EL UK

**Keywords:** Antibody therapy, Predictive markers, Neuroscience, Mechanisms of disease

Correction to: *Eye* 10.1038/s41433-021-01807-4, published online 05 January 2022

In the first published version of this article, the corresponding author was listed incorrectly. The corresponding author is only Giulia De Rossi, e-mail: giulia.derossi@ucl.ac.uk

The original article has been corrected

